# High social risk and mortality. A prospective study in community-dwelling older adults living in a rural Ecuadorian village

**DOI:** 10.1016/j.pmedr.2023.102146

**Published:** 2023-02-13

**Authors:** Oscar H. Del Brutto, Robertino M. Mera, Denisse A. Rumbea, Bettsy Y. Recalde, Mark J. Sedler

**Affiliations:** a School of Medicine and Research Center, Universidad Espíritu Santo – Ecuador, Samborondón, Ecuador; b Biostatistics/Epidemiology, Freenome, Inc., South San Francisco, CA, USA; c Renaissance School of Medicine, Stony Brook University, New York, NY, USA

**Keywords:** Social risk, Social determinants of health, Gijon’s social-familial evaluation scale, Mortality, Population-based cohort

## Abstract

High social risk has been associated with mortality, but information on this relationship in remote rural communities is limited. Using the social determinants of health (SDH) specified in the Gijon’s social-familial evaluation scale (SFES), we aimed to assess mortality risk according to levels of social risk in community-dwelling older adults living in rural Ecuador. Following a longitudinal population-based design, this study prospectively followed 457 individuals for an average of 8.2 ± 2.6 years. A total of 115 (25.2 %) individuals died during the study years. The mean Gijon’s SFES score was 9.4 ± 2.8 points among survivors *versus* 12.3 ± 4 points among those who died (*p* < 0.001). Separate models using individual SDH components as exposures showed that deficits in family situation, social relationships and support networks were significantly associated with mortality, whereas economic status and housing factors were not. A Cox-proportional hazard model, with the Gijon’s SFES score stratified in tertiles, showed a more than 5-fold increase in mortality among individuals in the third tertile compared with those in first and second tertiles, after adjusting for relevant covariates (HR: 5.36; 95 % C.I.: 3.09 – 9.32). Study results indicate an important contribution of high social risk to mortality, and may help to identify potential interventional targets that are focused on encouraging social interactions, and that may reduce mortality in older adults living in remote settings.

## Highlights

• High social risk has been associated with premature mortality in people living in urban centers.
• Information on this relationship in remote rural communities is limited.
• Individuals with high social risk have 5-fold increased mortality after more than eight years of follow-up.
• Social isolation was the most important component in the above-mentioned relationship.

## 1 Introduction

The conditions in which people are born, grow up, work, live and come of age are often referred as the social determinants of health (SDH). These determinants include their living environment, quality of education, economic stability, food security, social relationships, support networks, and access to healthcare and are useful in measuring the social risk of individuals (Marmot et al., 2008). SDH have a tremendous impact on the wellbeing of individuals as high social risk may foster the occurrence or progression of several diseases (Cockerham et al., 2017, Ferrario et al., 2017, Reshetnyak et al., 2020, Walker et al., 2016). In addition, high social risk has been associated with increased mortality independent of the presence of other risk factors or morbidities (Checa et al., 2019, Chu et al., 2021, Henriksen et al., 2019, Holseter et al., 2015, Holwerda et al., 2012).

While the relationship between social risk and mortality has been extensively studied in urban centers of High-income countries, data from Low- and Middle-income countries are limited and what findings do exist are heterogeneous due to differences in the components of SDH that were used to estimate the social risk in these disparate populations (González-Bautista et al., 2019, Ibrahim, 2016, Zeng et al., 2010).

The evaluation of social risk should be tailored according to the characteristics of a given community and the interpretation of that evaluation must take account of these differences in makeup (Bourne, 2009, Riva et al., 2011). Race/ethnicity, poverty, illiteracy, and disparities in healthcare access have been recognized as important SDH in high income settings (Sacco, 2020). However, these determinants are less significant among people living in poor-resource rural settings, a fact that highlights the importance of selecting an appropriate field instrument suited to the investigation of SDH in rural, remote, and underserved populations.

Utilizing the Atahualpa Project cohort, this longitudinal study aims to assess the relationship between social risk – as measured by the SDH – and mortality in community-dwelling older adults living in rural Ecuador.

## 2 Methods

**Study population:** This study was conducted in community-dwellers aged ≥ 60 years living in Atahualpa, a rural Ecuadorian village where previous cross-sectional studies on clinical and neuroimaging correlates of high social risk have been carried out (Del Brutto et al., 2022a, Del Brutto et al., 2022b). As detailed elsewhere, the population is homogeneous regarding ethnicity (Amerindian ancestry), low education levels, middle-to-low income, and a diet rich in oily fish and carbohydrates but poor in other types of meat and dairy products (Del Brutto et al, 2018). Atahualpa has a minimal migration rate, which makes the village an optimal setting for the realization of prospective longitudinal studies.

**Study design:** Following a longitudinal prospective design, Atahualpa residents aged ≥ 60 years were identified by means of annual door-to-door surveys (from June 2012 to June 2019) and those who received SDH determinations in the year after enrolment were included in the study. All participants (or their legal representatives in case of disability) signed a comprehensive informed consent form before entering the study. The population was closely followed during the study years, and our field personnel continuously reminded participants’ proxies to notify us in the case of death.

The last censoring date was set as of July 2022. Those who emigrated or declined consent were removed from the cohort at the administrative censoring date of the last annual survey in which the individual was interviewed. Individuals who died were censored at the time of death. The study was approved by the Ethics Committee of Hospital-Clínica Kennedy, Guayaquil (FWA 00030727). Aggregated data from this study are available from the corresponding author upon reasonable request.

**Social risk assessment:** Social risk was determined using the Gijon’s Social-Familial Evaluation Scale (SFES) (Cabrera González et al., 1999). This validated field instrument (originally developed in Spanish) rates five SDH including family situation, economic status, housing, social relationships and support networks. Each of these components has five questions weighted on a 1 to 5 scale, for a maximal score of 25, with greater scores indicating higher social risk (Cabrera González et al., 1999). The Gijon’s SFES was selected due to its suitability to the living conditions of the study population and because it has been endorsed by the Ecuadorian Minister of Health for the assessment of social risk (Ministerio de Salud Pública del Ecuador, 2008).

**Covariates:** Demographics (age and sex), level of education (primary school education or higher), traditional cardiovascular risk factors, and history of an overt stroke were selected as relevant covariates. Criteria proposed by the American Heart Association (AHA) were used to assess cardiovascular risk factors as follows: a poor smoking status was designated if the subject was a current smoker or quit < 1 year, a poor body mass index if ≥ 30 kg/m^2^, a poor physical activity if there was no moderate or vigorous activity, a poor diet if the individual had 0–1 component of the AHA healthy diet, a poor blood pressure if ≥ 140/90 mmHg, a poor fasting glucose if ≥ 126 mg/dL, and a poor total cholesterol blood level if ≥ 240 mg/dL (Lloyd-Jones et al., 2010). A potential stroke at baseline was identified by field personnel interviews with patients or their proxies using a previously validated field instrument (Del Brutto et al., 2004), and the diagnosis was confirmed by a certified neurologist with the aid of a brain MRI.

**Statistical analysis:** All data analyses were carried out by using STATA version 17 (College Station, TX, USA). Continuous variables were compared by linear models and categorical variables by the chi-square or Fisher exact test in unadjusted analyses. To compute the person-years of follow-up we considered the time from enrollment to the last censoring date, study drop-out, or death. Cox-proportional hazard models were fitted to calculate the hazard ratio (HR) with its 95 % confidence interval (CI) in order to estimate mortality risk (outcome) according to the Gijon’s SFES total score and of each of its components, after adjusting for age, sex, level of education, cardiovascular risk factors, and history of overt strokes at baseline.

## 3 Results

Of 478 Atahualpa residents aged ≥ 60 years enrolled in the Atahualpa Project cohort, 457 (96 %) had SDH determinations and were included in this cohort. The remaining 21 individuals either died (n = 11) between enrollment and the invitation to participate in this study or declined consent (n = 10). The total follow-up was 3,752 person-years and the average follow-up was 8.2 ± 2.6 years (median: 10.1 years; interquartile range: 6.6 – 10.1 years). Individuals censored before the end of the study were those who emigrated (n = 15), declined further consent (n = 34), or died (n = 115) during the study years; however, they also counted toward the total time of follow-up.

The mean (±SD) age of study participants at enrollment was 62.2 ± 8.4 years (median age: 66 years), 256 (56 %) were women, and 353 (77 %) had primary school education only. Fourteen (3 %) individuals were smokers, 106 (23 %) were obese, 54 (12 %) reported poor physical activity, 20 (4 %) had an unhealthy diet, 208 (46 %) had high blood pressure, 146 (32 %) had high fasting glucose, 55 (12 %) had high total cholesterol levels, and 37 (8 %) had history of a stroke at baseline.

The mean (±SD) Gijon’s SFES score was 10.1 ± 3.1 points with a median of 9 points. Mean scores of components of this scale were as follows: Family situation: 1.9 ± 1.2 points; Economic status: 3.2 ± 0.9 points; Housing: 1.8 ± 0.8 points; Social relationships: 1.8 ± 1.1 points; and Support networks: 1.5 ± 0.9 points. Higher scores in the Gijon’s SFES stratified in tertiles (5–8, 9–11, and ≥ 12 points) were noticed among older and less educated individuals as well as in those with poor physical activity and with history of an overt stroke at baseline (Table 1). Also in unadjusted analyses, mean values of the total Gijon’s SFES score, as well as three of its components (family situation, social relationships and support networks) were higher among individuals who died during the study years compared to those who survived and were administratively censored (Table 2).

**Table 1:** Characteristics of 457 community-dwelling older adults enrolled in this study according to tertiles of the Gijon’s Social Familial Evaluation Scale (unadjusted analyses).

Variable	Gijon’s Social Familial Evaluation Scale			*p* value
	Tertile 1 (5–8 points) n = 168	Tertile 2 (9–11 points) n = 157	Tertile 3 (≥12 points) n = 132	
**Age (in years) at baseline, mean ± SD.**	65.3 ± 6.5	67.1 ± 7.3	73.2 ± 9.5	<0.001*
**Women, n (%)**	95 (57)	82 (52)	79 (60)	0.423
**Primary school education, n (%)**	110 (65)	123 (78)	120 (91)	<0.001*
**Current smoker, n (%)**	7 (4)	4 (3)	3 (2)	0.575
**Body mass index ≥ 30 kg/m^2^, n (%)**	44 (26)	34 (22)	28 (21)	0.510
**Poor physical activity, n (%)**	9 (5)	14 (9)	31 (23)	<0.001*
**Poor diet, n (%)**	3 (2)	11 (7)	6 (5)	0.071
**Blood pressure ≥ 140/90 mmHg, n (%)**	70 (42)	67 (43)	71 (54)	0.076
**Fasting glucose ≥ 126 mg/dL, n (%)**	44 (26)	50 (32)	52 (39)	0.052
**Total cholesterol ≥ 240 mg/dL, n (%)**	26 (15)	16 (10)	13 (10)	0.225
**Overt stroke at baseline, n (%)**	7 (4)	11 (7)	19 (14)	0.005*

*Statistically significant result.

**Table 2:** Social determinants of health, as measured by the Gijon’s Social-Familial Evaluation Scale, according to vital status at censoring date (unadjusted analysis).

Variable	Total series (n = 457)	Vital status at censoring date		*p* value
		Alive (n = 342)	Dead (n = 115)	
**Gijon’s SFES score, mean ± SD**	10.1 ± 3.1	9.4 ± 2.8	12.3 ± 3	<0.001*
**Family situation, mean ± SD**	1.9 ± 1.2	1.7 ± 1.1	2.5 ± 1	<0.001*
**Economic status, mean ± SD**	3.2 ± 0.9	3.2 ± 1	3.3 ± 0.9	0.342
**Housing, mean ± SD**	1.8 ± 0.8	1.8 ± 0.9	1.7 ± 0.7	0.278
**Social relationships, mean ± SD**	1.8 ± 1.1	1.5 ± 0.9	2.6 ± 1	<0.001*
**Support networks, mean ± SD**	1.5 ± 0.9	1.2 ± 0.6	2.3 ± 1.1	<0.001*

*Statistically significant result; SFES: Social-familial evaluation scale.

**Fig. 1:**
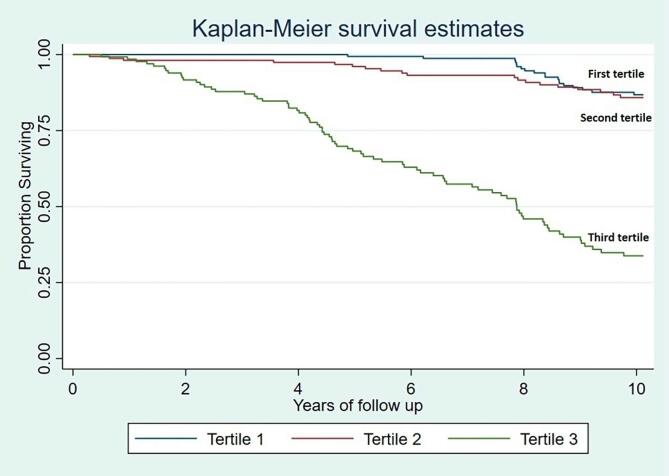
Kaplan-Meier survival curves and Hazards Ratios with 95% confidence intervals for all-cause mortality according to levels of social risk (categorized according to the Gijon’s social-familial evaluation scale). The Cox-proportional hazards model shows a more than 5-fold increased mortality among individuals in the third tertile of the Gijon’s social-familial evaluation scale compared with those in first and second tertiles.

A Cox-proportional hazard model, with the Gijon’s SFES score stratified in tertiles, showed increased mortality among individuals in the third tertile compared with those in first and second tertiles (Fig. 1). Likewise, a fully-adjusted Cox-proportional hazard model showed a more than 5-fold increased mortality (HR: 5.36; 95 % C.I.: 3.09 – 9.32) among individuals in the third tertile of the Gijon’s SFES score compared with those in the first and second tertiles (Table 3). Covariates remaining independently significant in this model included increased age at baseline, being male, having poor physical activity, and high fasting glucose.

**Table 3:** Fully-adjusted Cox-proportional hazard model showing a more than 5-fold increased mortality among individuals in the third tertile of the Gijon’s SFES score compared with those in the first and second tertiles.

Mortality	HR	95 % C.I.	*p* value
**First tertile of the Gijon’s SFES score**	Referent category		
**Second tertile of the Gijon’s SFES score**	0.98	0.51 – 1.86	0.948
**Third tertile of the Gijon’s SFES score**	5.51	3.18 – 9.55	<0.001*
**Age at baseline**	1.06	1.03 – 1.08	<0.001*
**Being female**	0.63	0.43 – 0.94	0.022*
**Primary school education**	0.72	0.42 – 1.25	0.243
**Current smoker**	0.94	0.28 – 3.09	0.915
**Body mass index ≥ 30 kg/m^2^**	0.92	0.56 – 3.09	0.915
**Poor physical activity**	1.91	1.17 – 3.12	0.009*
**Poor diet**	1.51	0.67 – 3.37	0.319
**Blood pressure ≥ 140/90 mmHg**	0.84	0.57 – 1.24	0.378
**Fasting glucose ≥ 126 mg/dL**	1.62	1.09 – 2.41	0.018*
**Total cholesterol ≥ 240 mg/dL**	0.68	0.34 – 1.37	0.281
**Overt stroke (baseline and follow-up)**	1.12	0.64 – 1.97	0.689

*Statistically significant result.

SFES: Social-familial evaluation scale.

## 4 Discussion

This longitudinal cohort study demonstrated a significant association between high social risk and mortality. Three components of SDH – family situation, social relationships and support networks – had an independent relationship with mortality. The other two components of the Gijon’s SFES did not influence the vital status at follow-up likely because economic status and housing conditions were relatively similar among all Atahualpa residents included in this cohort.

None of the investigated covariates tempered the significance of the above-mentioned relationships, suggesting that the impact of high social risk on death surpassed that of increasing age, sex, level of education, vascular risk factors, and history of overt stroke.

Our results are consistent with previous studies that show social isolation is associated with an increased risk of mortality among older adults (Checa et al., 2019, Henriksen et al., 2019, Holseter et al., 2015, Holwerda et al., 2012). Indeed, a metanalysis of prospective studies assessing mortality risk associated with SDH components of social relationships and support networks revealed that social isolation, feelings of loneliness and living alone accounted for 29 %, 26 % and 32 % increased mortality, respectively (Holt-Lundstad et al., 2015).

Pathogenic mechanisms explaining the link between social risk and mortality risk are not yet well understood. A recent review elaborates on the contributory role of high social risk in the occurrence of ailments and mortality (Bhatti & Haq, 2017). According to the reviewed literature, high social risk increases the risk of cardiovascular, metabolic, inflammatory and other conditions that, in turn, make individuals more likely to die. However, the effect of high social risk supersedes those factors, and it is possible that social isolation reduces the resilience of individuals in coping with illness.

This study has some limitations. SDH and vascular risk factors were measured at baseline, but they may have changed during follow-up. However, the study population has been closely followed and most participants underwent repeated determinations of vascular risk factors during the study years. We did not identify significant changes in vascular risk factors with the exception of a temporary decline in physical activity and dietary habits during the past two years as a result of the SARS-CoV-2 pandemic (Del Brutto et al., 2021). The study included a homogeneous population of older adults of Amerindian ancestry living in a remote rural village, and, therefore, our results may not be generalizable to other races/ethnic groups or to people living in other settings. It is also possible that some unmeasured confounders are responsible for at least part of the findings of the present study. In addition, suspected inaccuracies in death certificates and verbal autopsy reports limited the correct classification of cause of death. These limitations are balanced by the study’s strengths that include a population-based design, the systematic assessment of vascular risk factors and stroke by means of uniform protocols, and the determination of social risk by means of a field instrument that adjusts to the characteristics of the study population. In addition, a power analysis showed that with a median follow-up of about 10 years, in a population of 457 subjects and an overall expected mortality proportion of ∼ 25 %, the expected number of events (death) was ∼ 110 over the period of observation. Fixing significance at the 0.05 level, there was more than 80 % power to detect a difference provided by a Hazard Ratio larger than 1.7.

Understanding the burden of social risk is important for public health planning and the development of cost-effective preventive policies aimed at reducing mortality in vulnerable populations. Intervention strategies may include facilitating group activities and the reinforcement of social networks among older adults living in rural settings. These activities should be personalized according to the social needs of the population and can be accomplished by social workers or by community members trained in these tasks. However, the parameters and duration of such interventions that may be needed in order to positively influence adverse outcomes remain to be ascertained. Even so, the results of the present study open avenues of research to better understand the pathogenic mechanisms involved in the relationship between high social risk and mortality.

## 5 Conclusion

High social risk is an independent predictor of mortality in older adults living in rural settings. SDH components related to social isolation – poor family situations, poor social relationships and deficient support network – account for deadly outcomes. This study adds to the accumulating evidence of the impact of social risk on mortality, and suggests interventional targets focused on encouraging social interactions that may prove to be cost-effective for reducing mortality in vulnerable populations. In the same way that smoking, obesity and arterial hypertension receive conspicuous media coverage, the deleterious consequences of high social risk, and in particular social isolation should be given more attention as an opportunity for community-based preventive public health programs.

## 6 Source of funding

Universidad Espiritu Santo – Ecuador, Samborondón, Ecuador.

## Declaration of Competing Interest

The authors declare that they have no known competing financial interests or personal relationships that could have appeared to influence the work reported in this paper.

## Data Availability

Data will be made available on request.
